# Venous Thrombosis Among Critically Ill Patients With Coronavirus Disease 2019 (COVID-19)

**DOI:** 10.1001/jamanetworkopen.2020.10478

**Published:** 2020-05-29

**Authors:** Julien Nahum, Tristan Morichau-Beauchant, Fabrice Daviaud, Perrine Echegut, Jérôme Fichet, Jean-Michel Maillet, Stéphane Thierry

**Affiliations:** 1 Intensive Care Unit, Centre Cardiologique du Nord, Saint-Denis, France; 2 Ultrasound and Vascular Lab, Centre Cardiologique du Nord, Saint-Denis, France

## Abstract

This case series reports a systematic assessment of deep vein thrombosis among patients in an intensive care unit in France with severe coronavirus disease 2019 (COVID-19).

## Introduction

Severe acute respiratory syndrome coronavirus 2 (SARS-CoV 2) was identified as a new coronavirus causing pneumonia and acute respiratory distress syndrome. It has become a pandemic, spreading particularly quickly across Europe and the US. Most deaths are related to severe acute respiratory distress syndrome, but other organ failures, such as acute kidney failure and acute cardiac injury, seem also related to the disease.^1^ Inflammatory response is highly increased in coronavirus disease 2019 (COVID-19) infection, and inflammation is known to favor thrombosis. High dimerized plasmin fragment D (D-dimer) levels and procoagulant changes in coagulation pathways were reported among patients with severe COVID-19.^2,3^ An elevated rate of venous and arterial thrombotic events associated with COVID-19 infection has also been reported.^4,5^ This case series reports a systematic assessment of deep vein thrombosis among patients in an intensive care unit (ICU) in France with severe COVID-19.

## Methods

This case series was approved by the ethical committee of the Centre Cardiologique du Nord, which granted a waiver of consent because the research presented no risk of harm and required no procedures for which consent is normally required outside a research context. This study followed the Strengthening the Reporting of Observational Studies in Epidemiology (STROBE) reporting guideline. Patients with severe COVID-19 pneumonia were admitted to our ICU located in the suburban Paris area from mid-March 2020 to the beginning of April 2020. All patients had acute respiratory distress syndrome according to the Berlin definition and required mechanical ventilation.

We prospectively performed a venous ultrasonogram of the inferior limbs for all patients at admission to our ICU, considering previous data that showed increased levels of inflammatory markers, preliminary reports from the intensive care community signaling frequent events of deep vein thrombosis in ICU patients with COVID-19 at the time we received our first patients, and the high rate of deep vein thrombosis found among the first patients with COVID-19 admitted to our unit. Considering the high prevalence of venous thrombosis at admission, we systematically repeated venous ultrasonography after 48 hours if the first examination returned normal results. As recommended, all patients received anticoagulant prophylaxis at hospital admission. Statistical analyses were conducted in Prism version 5.0 (GraphPad) and Excel 365 (Microsoft Corp). Statistical significance was set at *P* < .05, and all tests were 2-tailed.

## Results

A total of 34 consecutive patients were included in this study. COVID-19 diagnosis was confirmed with polymerase chain reaction on nasopharyngeal swabs of 26 patients (76%); 8 patients (24%) had a negative result on polymerase chain reaction but had a typical pattern of COVID-19 pneumonia on chest computed tomography scan. Mean (SD) age was 62.2 (8.6) years, and 25 patients (78%) were men. Major comorbidities were diabetes (15 [44%]), hypertension (13 [38%]), and obesity (mean [SD] body mass index [calculated as weight in kilograms divided by height in meters squared], 31.4 [9.0]). Overall, 26 patients (76%) required norepinephrine at admission, 16 (47%) required prone positioning, and 4 (12%) required venovenous extracorporeal membrane oxygenation (Table 1). Only 1 patient (3%) received anticoagulant therapy before hospitalization.

**Table 1.** Clinical Characteristics and Laboratory Findings Among 34 Patients at Admission

Characteristic	Total	Patients without deep vein thrombosis	Patients with deep vein thrombosis
No. (%)	34 (100)	7 (21)	27 (79)
Age mean (SD), y	62.2 (8.6)	59.9 (11.2)	62.9 (7.9)
Men, No. (%)	25 (78)	5 (71)	20 (74)
BMI, mean (SD)	31.4 (9.0)	27.8 (4.8)	32.2 (9.6)
Comorbidities, No. (%)			
Cancer	1 (3)	0	1 (4)
Chronic obstructive pulmonary disease	2 (6)	1 (14)	1 (4)
Diabetes mellitus	15 (44)	3 (43)	12 (44)
Ischemic cardiopathy	3 (9)	2 (29)	1 (4)
ACE or ARB treatment	7 (21)	2 (29)	5 (19)
Hypertension	13 (38)	4 (57)	9 (33)
Time from hospital admission to ICU admission, mean (SD), d	1.6 (2.6)	2.7 (4.5)	1.3 (1.8)
Receipt of norepinephrine, No. (%)	26 (76)	5 (71)	21 (78)
Ventilation therapy, No. (%)			
Mechanical ventilation	34 (100)	7 (100)	27 (100)
Prone positioning	16 (47)	6 (86)	10 (37)
Nitric oxide inhalation	5 (15)	1 (14)	4 (15)
Venovenous ECMO	4 (12)	2 (29)	2 (7)
Laboratory findings, mean (SD)			
White blood cell count, /μL	9305 (3912)	7510 (3553)	9770 (3926)
Lymphocytes, /μL	1109 (566)	918 (731)	1158 (521)
Platelets, ×10^3^/μL	256 (107)	198 (106)	271 (104)
Serum creatinine, mg/dL	1 (0.5)	1.44 (0.99)	0.93 (0.25)
Prothrombin, % of activity	85 (11.4)	79.7 (16.1)	86.3 (9.7)
Activated clotting time, ratio	1.2 (0.1)	1.3 (0.1)	1.1 (0.1)
Fibrinogen, mg/dL	760 (170)	790 (150)	750 (180)
D-dimer level, mg/l	5.1 (5.4)	3.3 (2.6)	5.4 (5.8)
Troponin level, pg/ml	42.2 (57.2)	45.7 (56.5)	41.3 (58.4)
C-reactive protein, mg/dL	22.8 (12.9)	24.6 (16.0)	22.4 (12.3)
N-terminal pro–brain natriuretic peptide, pg/ml	518 (946)	251 (203)	602 (1072)

Abbreviations: ACE, angiotensin-converting enzyme; ARB, angiotensin receptor blockers; BMI, body mass index (calculated as weight in kilograms divided by height in meters squared); D-dimer, dimerized plasmin fragment D; ECMO, extracorporeal membrane oxygenation.

SI conversion factors: To convert C-creative protein to milligrams per liter, multiply by 10.0; creatinine to micromoles per liter, multiply by 88.4; D-dimer to nanomoles per liter, multiply by 5.476; fibrinogen to grams per liter, multiply by 0.01; lymphocytes and white blood cell count to ×10^9^ per liter, multiply by 0.001; n-terminal pro–brain natriuretic peptide to nanograms per liter, multiply by 1.0; platelets to ×10^9^ per liter, multiply by 1.0; and troponin to micrograms per liter, multiply by 1.0.

Deep vein thrombosis was found in 22 patients (65%) at admission and in 27 patients (79%) when the venous ultrasonograms performed 48 hours after ICU admission were included (Table 2). Eighteen patients (53%) had bilateral thrombosis, and 9 patients (26%) had proximal thrombosis. Comparable with previously published data,^2,3^ our population had high levels of D-dimer (mean [SD], 5.1 μg/mL [to convert to nanomoles per liter, multiply by 5.476]), fibrinogen (mean [SD], 760 [170] mg/dL [to convert to grams per liter, multiply by 0.01]) and C-reactive protein (mean [SD], 22.8 [12.9] mg/dL [to convert to milligrams per liter, multiply by 10]). Prothrombin activity (mean [SD], 85% [11.4%]) and platelet count (mean [SD], 256 [107] × 10^3^/μL) were normal (Table 1).

**Table 2.** Rate of Deep Vein Thrombosis at Admission and at 48 Hours

Deep vein thrombosis	Patients, No. (%) (N = 34)		
	At admission	48 h after admission	Total
Total	22 (65)	5 (15)	27 (79)
Proximal	8 (24)	1 (3)	9 (26)
Distal	19 (56)	4 (12)	23 (68)
Bilateral	15 (44)	3 (9)	18 (53)

## Discussion

Mortality of patients with COVID-19 admitted to ICUs has been reported to be high, at 50%.^6^ Frequent venous and arterial thrombotic events have been reported, with rates from 27% to 69% of peripheral venous thromboembolism and up to 23% of pulmonary embolism.^4,5^ The occurrence of pulmonary embolism might be favored by deep vein thrombosis. The main limitations of this study were its monocentric nature and the relatively small size of our cohort. In view of the high rate (ie, 79%) of deep vein thrombosis reported in this study, prognosis might be improved with early detection and a prompt start of anticoagulant therapy. Despite anticoagulant prophylaxis, 15% of our patients developed deep vein thrombosis only 2 days after ICU admission. Systematic anticoagulant therapy for all ICU patients with COVID-19 should be assessed.
